# Culture-Based Virus Isolation To Evaluate Potential Infectivity of Clinical Specimens Tested for COVID-19

**DOI:** 10.1128/JCM.01068-20

**Published:** 2020-07-23

**Authors:** Chung-Guei Huang, Kuo-Ming Lee, Mei-Jen Hsiao, Shu-Li Yang, Peng-Nien Huang, Yu-Nong Gong, Tzu-Hsuan Hsieh, Po-Wei Huang, Ya-Jhu Lin, Yi-Chun Liu, Kuo-Chien Tsao, Shin-Ru Shih

**Affiliations:** a Department of Laboratory Medicine, Linkou Chang Gung Memorial Hospital, Taoyuan, Taiwan; b Department of Medical Biotechnology and Laboratory Science, College of Medicine, Chang Gung University, Taoyuan, Taiwan; c Research Center for Emerging Viral Infections, College of Medicine, Chang Gung University, Taoyuan, Taiwan; d Division of Infectious Diseases, Department of Pediatrics, Linkou Chang Gung Memorial Hospital, Taoyuan, Taiwan; e Research Center for Chinese Herbal Medicine, Research Center for Food and Cosmetic Safety, and Graduate Institute of Health Industry Technology, College of Human Ecology, Chang Gung University of Science and Technology, Taoyuan, Taiwan; Boston Children's Hospital

**Keywords:** RT-PCR, SARS-CoV-2, culturability, genome copy, genome integrity

## Abstract

Real-time reverse transcription-PCR (RT-PCR) is currently the most sensitive method to detect severe acute respiratory syndrome coronavirus 2 (SARS-CoV-2) that causes coronavirus disease 2019 (COVID-19). However, the correlation between detectable viral RNA and culturable virus in clinical specimens remains unclear. Here, we performed virus culture for 60 specimens that were confirmed to be positive for SARS-CoV-2 RNA by real-time RT-PCR. The virus could be successfully isolated from 12 throat and nine nasopharyngeal swabs and two sputum specimens. The lowest copy number required for virus isolation was determined to be 5.4, 6.0, and 5.7 log_10_ genome copies/ml sample for detecting the *nsp12*, *E*, and *N* genes, respectively. We further examined the correlation of genome copy number and virus isolation in different regions of the viral genome, demonstrating that culturable specimens are characterized by high copy numbers with a linear correlation observed between copy numbers of amplicons targeting structural and nonstructural regions. Overall, these results indicate that in addition to the copy number, the integrity of the viral genome should be considered when evaluating the infectivity of clinical SARS-CoV-2 specimens.

## Abstract (precis)

Real-time reverse transcription-PCR (RT-PCR) is currently the most sensitive method to detect severe acute respiratory syndrome coronavirus 2 (SARS-CoV-2) that causes coronavirus disease 2019 (COVID-19). However, the correlation between detectable viral RNA and culturable virus in clinical specimens remains unclear. Here, we performed virus culture for 60 specimens that were confirmed to be positive for SARS-CoV-2 RNA by real-time RT-PCR. The virus could be successfully isolated from 12 throat and nine nasopharyngeal swabs and two sputum specimens.

## INTRODUCTION

The pandemic of severe acute respiratory syndrome coronavirus 2 (SARS-CoV-2) that is the cause of the respiratory disease coronavirus disease 2019 (COVID-19) has resulted in tens of thousands of deaths globally since it was first identified in Wuhan, China, at the end of 2019 (1, 2). Clinical manifestations of COVID-19 range from mild symptoms to severe illness and even death. Most patients develop respiratory symptoms such as fever, cough, and shortness of breath (3). Other nonrespiratory symptoms, including diarrhea, anosmia, and neurological and myocardial injuries, have also been reported despite the uncertain etiology (4). A broad tissue tropism and transmissibility have been proposed for SARS-CoV-2 based on sequence comparison combined with structure analyses of the viral spike protein (5–7). Viral RNA is detectable not only from respiratory specimens but also in the urine, serum, and stool using reverse transcription-PCR (RT-PCR), and viral RNA has been detected in COVID-19 patients for more than 30 days (8–13). However, no virus has been isolated from either stool or respiratory specimens collected after day 8 of illness, even in samples with a high viral RNA concentration (9). Thus, nucleic acid detection by RT-PCR requires validation by additional assays such as labor-intensive culture-based virus isolation to assess the extent of virus shedding or infectiousness of the specimens (14).

To clarify the correlation between the culturability of the virus from clinical specimens and the RNA copy number, in the present study, we investigated the culturability of a total of 60 specimens from 50 laboratory-confirmed COVID-19 patients that were collected from 25 January to the end of March 2020 in Taiwan. We assessed the association between the cycle threshold (*C_T_*) value and RNA levels for genes encoding RNA-dependent RNA polymerase (*nsp12*), envelope (*E*), and nucleocapsid (*N*) proteins according to World Health Organization guidelines (15) from respiratory specimens of the throat, including oropharyngeal (OP) and nasopharyngeal (NP) swabs, or sputum (SP). These findings can provide relevant practical insight for determining the infectivity of clinical specimens toward helping to control the spread of this virus and curb the current pandemic.

## MATERIALS AND METHODS

### Ethics statement.

This study was approved by the Institutional Review Board of Chang Gung Medical Foundation, Linkou Medical Center, Taoyuan, Taiwan (approval no. 202000468B0B1).

### RT-PCR analysis of samples from confirmed COVID-19 patients.

As a reference laboratory of the Taiwan Centers for Disease Control (CDC; https://www.cdc.gov.tw/En/), we have been performing viral diagnosis for suspected COVID-19 patients. Specimens of suspected COVID-19 cases collected by border control systems and different hospitals around Taipei county are routinely sent to our clinical virology laboratory. This study included 60 specimens from 50 cases. Respiratory specimens of the OP swab or NP swab and/or sputum were collected depending on the sample availability for each case/patient, and specimen sampling and transportation were handled according to the criteria of the Taiwan CDC. All respiratory samples were maintained in a universal transport medium (UTM-RT; Copan Diagnostics) for further analysis. SARS-CoV-2 nucleic acids were detected by real-time RT-PCR according to the guidelines of the Taiwan CDC. In brief, RNA was extracted from clinical specimens by the automatic LabTurbo system (Taigen, Taiwan) following the manufacturer’s instructions for the most part, except that the specimen was pretreated with proteinase K prior to RNA extraction. Reagents and primer/probe sets used to respectively detect *E*, *N*, and *nsp12* RNA were described by Corman et al. (15), and RT-PCR was performed in a 25-μl reaction mixture containing 5 μl of RNA.

### Calculation of the genome copy number from the *C_T_* value.

SARS-CoV-2 cDNA was prepared using RNA extracted from the specimens of the first patient with confirmed COVID-19. RT was performed using the Moloney murine leukemia virus (MMLV) reverse transcription kit (Protech, Taiwan) according to the manufacturer’s instructions. Amplified *E*, *N*, and *nsp12* cDNA was subsequently cloned into the pCRII-TOPO vector (Thermo Fisher Scientific, Waltham, MA, USA) in antisense orientation. *In vitro* transcription using the linearized plasmid as the template to synthesize *E*, *N*, and *nsp12* RNA was performed as described by Lee et al. (16). Purified RNA was then quantified by a Qubit fluorometer (Thermo Fisher Scientific), and serially diluted standard RNAs were prepared for subsequent real-time RT-PCR (15). The primer sequences used to amplify the *E*, *N*, and *nsp12* genes were as follows: SARS-CoV-2-E-For, 5′-ATGTACTCATTCGTTTCGGAAGAGAC-3′; SARS-CoV-2-E-Rev, 5′-TTAGACCAGAAGATCAGGAACTCTAG-3′; SARS-CoV-2-N-For, 5′-ATGTCTGATAATGGACCCCAAAATCAGC-3′, SARS-CoV-2-N-Rev, 5′-TTAGGCCTGAGTTGAGTCAGCACTGCTC-3′; SARS-CoV-2-nsp12-For, 5′-ATGCTTCAGTCAGCTGATGCACAATCGT-3′; and SARS-CoV-2-nsp12-Rev, 5′-CTGTAAGACTGTATGCGGTGTGTACATA-3′.

### Culture-based virus isolation.

All procedures for viral culture followed the laboratory biosafety guidelines of the Taiwan CDC and were conducted in a biosafety level 3 facility. Vero-E6 (American Type Culture Collection [ATCC], Manassas, VA, USA) and MK-2 (ATCC) cells were maintained in modified Eagle’s medium (MEM; Thermo Fisher Scientific) supplemented with 10% fetal bovine serum and 1× penicillin-streptomycin at 37°C in the presence of 5% CO_2_. Viral culture was initiated from standard screw-cap culture tubes (16 × 125 mm; Thermo Fisher Scientific), and cells grown to 80 to 90% confluence were inoculated with 500 μl of the virus solution containing 33 μl of the specimen and 2× penicillin-streptomycin solution for absorption at 37°C for 1 h. Subsequently, 5 ml of the virus culture medium composed of MEM, 2% fetal bovine serum, and 1× penicillin-streptomycin solution was added to the tubes, and the cells were maintained in a 37°C incubator with daily observations of the cytopathic effect. RT-PCR analysis was performed using the RNA extracted from the culture supernatant every 2 days after the initial inoculation to validate the presence of SARS-CoV-2.

### Statistical analysis.

The chi-square test was used to compare the culture rate of specimens that were subjected to a freeze cycle and those that were not. Student’s *t* test was used to analyze the differences in culture days required and RT-PCR results. Both analyses were performed using GraphPad Prism 7.00 (GraphPad Software, Inc., CA, USA) to compare the means for two groups. Data were presented as the mean ± SEM, and *P* < 0.05 was considered to indicate a statistically significant difference. Linear regression models were used to determine the correlation between the genome copies of structural and nonstructural genes with *C_T_* values from RT-PCR, and the *R*^2^ value was used to assess model fitness. This statistical analysis was conducted using R software (version 3.6.1) (17), and the distributions of genome copies and their correlations were visualized using the R package ggplot2 (18).

## RESULTS

### Isolation of SARS-CoV-2 from respiratory specimens.

Among the 60 specimens analyzed in this study from 50 cases, cases 3, 4, 6 to 10, and 12 to 15 were from a cluster infection at a single hospital, and cases 27 and 49 were from a household cluster; the *C_T_* values of each gene from individual specimens are listed in Table S1 in the supplemental material. Specimens collected before March (16 of the 60) were stored at −70°C until the SARS-CoV-2 isolation procedures obtained certification from the Taiwan CDC. Starting in March, virus culture was attempted on all specimens without a freeze-thaw cycle. We successfully obtained 23 isolates from different specimen types (12 from OP, nine from NP, and two from SP). We also obtained five isolates among the 16 specimens that underwent a single freeze-thaw cycle, although a significantly longer culture time was required compared to that of non-freeze-thaw specimens (13.8 ± 1.91 and 4.28 ± 0.39 days, respectively; *P* < 0.0001). The culture rate was low (3/19, 16%) for samples from patients who were characterized by a longer duration between the date of symptom onset and sample collection. Overall, our results suggested that a freeze-thaw cycle might not significantly affect the culture rate as previously described (19), with a success rate of 31% (5/16) obtained for the freeze-thaw samples compared to 41% (18/44) for the others (chi-square statistic 0.2136, *P* value, 0.6440; not significant [ns]). However, multiple freeze-thaw cycles should be prevented, because a significantly longer culture time was required for specimens subjected to a freeze-thaw cycle, which might disrupt the integrity of the virus and decrease its infectivity. In addition, the sample collection time might be a determinant in culturability, as specimens collected closer to the start of the illness date tended to be more culturable.

### Association of culturable samples with *C_T_* value.

**FIG 1:**
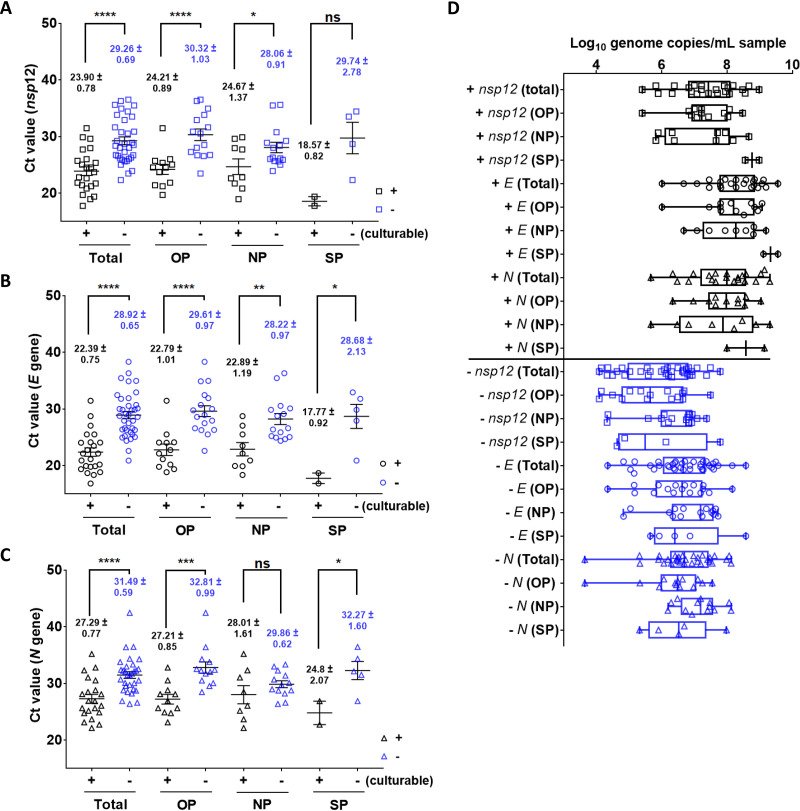
(A to C) Distribution of cycle threshold (*C_T_*) values and genome copies of the *nsp12* (A), *E* (B), and *N* (C) SARS-CoV-2 genes in culturable (black) and nonculturable (blue) specimens. Data are presented as means ± SEM (****, *P* < 0.0001; ***, *P* < 0.001; **, *P* < 0.01; *, *P* < 0.1; ns, not significant). (D) A box plot shows the calculation of log_10_ genome copies/ml from the *C_T_* value, including the minimum to maximum values. The median is depicted as a line within the box.

We next compared the RT-PCR results of the culturable and nonculturable specimens. The mean *C_T_* values for the *nsp12*, *E*, and *N* genes from all specimens and for each type of specimen (OP, NP, and SP) are summarized in Table 1. For all specimen types, the culturable specimens were characterized by a significantly lower *C_T_* value for all three genes (Fig. 1A to C), and the highest *C_T_* value that was sufficient for virus isolation was determined to be 31.47, 31.46, and 35.2 for the *nsp12*, *E*, and *N* genes, respectively (Table 1). We further compared the *C_T_* values of different specimen types. Regarding the *nsp12* and *E* genes, the mean *C_T_* value of culturable OP and NP specimens was similar to that of the total group (Fig. 1A and B). However, the culturable SP specimens were associated with much lower *C_T_* values despite the small number of cases analyzed for this group. Interestingly, differences between *C_T_* values of the *N* gene were clearly detected between the culturable and nonculturable specimens for the total, OP, and SP groups; however, no significant difference was detected for NP specimens (Fig. 1C). These results suggested that culturable specimens are characterized by a lower *C_T_* value in RT-PCR analysis, indicating the presence of more viral RNAs that allow for obtaining more virus isolates for culture.

**TABLE 1:** Cycle threshold values and genome copy numbers of three SARS-CoV-2 genes from specimens with and without isolation of the virus

Gene	Sample (*n*)	*C_T_* value			Log_10_ genome copies/ml		
		Mean ± SEM	Highest	Lowest	Mean ± SEM	Highest	Lowest
Culturable (*n* = 23)							
*nsp12*	Total (23)	23.90 ± 0.78	31.47	17.75	7.37 ± 0.20	8.98	5.40
	OP (12)	24.21 ± 0.89	31.47	19.69	7.29 ± 0.23	8.47	5.40
	NP (9)	24.67 ± 1.37	29.87	18.94	7.17 ± 0.36	8.67	5.82
	SP (2)	18.57 ± 0.82	19.38	17.75	8.76 ± 0.21	8.98	8.55
*E*	Total (23)	22.39 ± 0.75	31.46	16.85	8.21 ± 0.18	9.55	6.01
	OP (12)	22.79 ± 1.01	31.46	18.85	8.11 ± 0.24	9.07	6.01
	NP (9)	22.89 ± 1.19	28.74	18.36	8.09 ± 0.28	9.19	6.67
	SP (2)	17.77 ± 0.92	18.68	16.85	9.33 ± 0.22	9.55	9.11
*N*	Total (21)	27.29 ± 0.77	35.20	22.14	7.87 ± 0.21	9.30	5.67
	OP (11)	27.21 ± 0.85	32.81	23.13	7.89 ± 0.24	9.03	6.33
	NP (8)	28.01 ± 1.61	35.20	22.14	7.67 ± 0.45	9.30	5.67
	SP (2)	24.8 ± 2.07	26.86	22.73	8.56 ± 0.58	9.14	7.99
Nonculturable (*n* = 37)							
*nsp12*	Total (34)	29.26 ± 0.69	36.52	22.32	5.98 ± 0.18	7.78	4.09
	OP (15)	30.32 ± 1.03	36.52	23.47	5.70 ± 0.27	7.49	4.09
	NP (15)	28.06 ± 0.91	35.60	23.92	6.29 ± 0.24	7.37	4.32
	SP (4)	29.74 ± 2.78	34.43	22.32	5.85 ± 0.72	7.78	4.63
*E*	Total (37)	28.92 ± 0.65	38.33	20.89	6.62 ± 0.16	8.57	4.34
	OP (17)	29.61 ± 0.97	38.33	22.61	6.46 ± 0.23	8.15	4.34
	NP (15)	28.22 ± 0.97	36.31	24.39	6.79 ± 0.24	7.72	4.83
	SP (5)	28.68 ± 2.13	32.93	20.89	6.68 ± 0.52	8.57	5.65
*N*	Total (31)	31.49 ± 0.59	42.47	26.39	6.70 ± 0.17	8.12	3.64
	OP (13)	32.81 ± 0.99	42.47	29.55	6.33 ± 0.28	7.54	3.64
	NP (13)	29.86 ± 0.62	33.34	26.39	7.15 ± 0.17	8.12	6.18
	SP (5)	32.27 ± 1.60	36.45	26.89	6.48 ± 0.45	7.98	5.32

### Genome copy requirement for virus isolation.

To better assess the viral load of the specimens, we evaluated the genome copy number for each gene using *in vitro*-synthesized *E*, *N*, and *nsp12* RNA as the standard. The detailed *C_T_* to genome copy conversion of each gene is shown in Table S2, and the converted genome copy numbers of the *nsp12*, *E*, and *N* genes of different specimens in the culturable and nonculturable groups are summarized in Table 1 and illustrated in Fig. 1D. Consistent with a recent finding reported by Wolfel et al. (9), the majority of the culturable specimens (20/23, 87% regarding *nsp12* gene) contained viral genome copy numbers higher than 6 log_10_ genome copies/ml sample (Fig. 1D, upper part). The lowest genome copy numbers of *nsp12*, *E*, and *N* were 5.4, 6.0, and 5.7 log_10_ genome copies/ml sample, respectively (Table 1). In contrast, although the estimated genome copy numbers of the nonculturable specimens were lower than 6 log_10_ genome copies/ml sample (Table 1), a certain portion of nonculturable specimens (13/34, 38% > 6.5; 4/34, 12% > 7 regarding *nsp12* gene) also had genome copy numbers near or higher than 7 log_10_ genome copies/ml sample (Fig. 1D, lower part). Thus, a threshold copy number required for virus isolation could not be defined. Nonetheless, the overall copy numbers were clearly higher in culturable specimens.

### Assessment of culturability based on genome integrity of the specimens.

**FIG 2:**
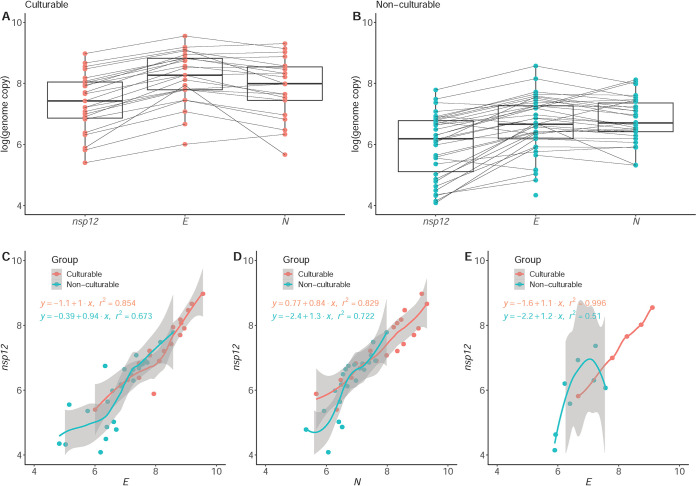
Distributions of genome copies in SARS-CoV-2 nonstructural (*nsp12*) and structural (*E* and *N*) genes of culturable (A) and nonculturable (B) specimens. Correlations of *nsp12* genome copies with those of the *E* (C) and *N* (D) genes in samples without a freeze-thaw cycle, and with *E* gene copy numbers in freeze-thawed samples (E), along with the respective regression equations and *R*^2^.

Since the *C_T_* value alone does not appear to be sufficient to determine whether the virus can be cultured from clinical specimens, we next tried to identify other parameters that might be used to assess infectivity. Coronavirus is characterized by a very large genome (∼30 kb) and a unique replication mechanism. Along with noncanonical RNAs, a total of 10 canonical RNAs composed of genomic and subgenomic RNAs are synthesized by multiple discontinuous transcription events during viral replication, including the *E* and *N* genes, which encode structural proteins and dominate the viral transcriptome (20, 21). Therefore, if signals of viral RNA detected in the clinical specimen originate from an intact genome, we would expect to observe a linear relationship between copies of nonstructural (*nsp12*) and structural (*E* and *N*) genes despite the use of different amplicons. The genome copy distributions of the *nsp12*, *E*, and *N* genes in culturable and nonculturable specimens are shown in Fig. 2A and B. Genome copies of individual genes in each of the specimens were connected, demonstrating higher expression of the *E* and *N* genes in both culturable and nonculturable samples. To avoid sampling bias, only samples that did not undergo a freeze-thaw cycle were first selected for analysis (Fig. 2C and D), showing a higher correlation between nonstructural and structural genes (*R*^2^ = 0.854 and 0.829 for *E* and *N*, respectively) in the culturable specimens than in the nonculturable specimens (*R*^2^ = 0.673 and 0.722, respectively). Moreover, the nonculturable specimens tended to contain additional copies of *E* and *N* RNAs. This disproportionate phenotype might be related to breakdown of the viral genome or contamination of subgenomic RNAs from host cells. To clarify these possibilities, we examined this correlation in specimens that underwent a freeze-thaw cycle (cases 1 to 12), since freeze-thaw might disrupt enveloped virus and genome integrity. Interestingly, as shown in Fig. 2E, we found a perfect correlation between the *nsp12* and *E* RNA copies in the culturable samples (*R*^2^ = 0.996) in sharp contrast to the nonculturable samples (*R*^2^ = 0.510). Further, the nonculturable samples were characterized by a markedly higher *nsp12* RNA level, suggesting the existence of degraded intermediates. Based on these findings, we speculated that nonculturable specimens containing higher or lower *nsp12* levels might reflect the detection of degraded genomes or replication intermediates, respectively. Therefore, monitoring the correlation of copy number among SARS-CoV-2 genes could be a useful parameter to determine whether the virus from a given specimen can be cultured.

## DISCUSSION

In this study, we investigated the infectivity of clinical specimens by virus culture and examined whether the infectivity was correlated with the level of viral nucleic acids. We provided quantitative results to show that specimens for which viral culture was successful contained more viral RNAs than those for which culture did not succeed. We also estimated the lowest genome copy numbers of specimens that would be sufficient for virus isolation. Since some nonculturable specimens also contained high genome copy numbers, the presence of viral nucleic acid alone cannot be used to assess the infectivity directly. By monitoring the correlation between amplicons targeting different genome loci, we found that examining the genome integrity might be another important criterion to evaluate the culturability/infectivity of clinical specimens. Although our conclusions are limited by the small sample size, potential sampling bias related to specimen collection and handling (e.g., source of specimen and timing), multiple types of storage and preservation before viral culture attempts, and lack of serial samples, these findings provide additional insight into assessing the infectiousness of COVID-19 patients.

Detection of SARS-CoV-2 by RT-PCR remains the gold standard test for confirming COVID-19, and two negative tests at least 24 h apart in a clinically recovered patient are one of the important criteria for hospital discharge as recommended by the World Health Organization (22). Viral shedding of SARS-CoV-2 has been estimated to occur more than 30 days after symptom onset (13, 23). Such prolonged and persistent detection of viral RNA in stool specimens, even after the negative conversion of respiratory specimens, led some researchers to suggest the potential for fecal-oral transmission of SARS-CoV-2 (10, 24), and the virus was recently proven to replicate in the human small intestinal epithelium and organoids (25). However, direct evidence for the infectiousness of these specimens collected long after symptom onset is lacking, and no virus culture from stool specimens has been achieved to date (9). Therefore, caution is needed when evaluating the infectivity of specimens simply based on the detection of viral nucleic acids (14). In the current study, we used a cell culture-based system to evaluate the infectivity. Several factors can affect virus isolation, including sampling bias, the cell line used, and the culture environment; however, culture remains the most reasonable approach to assess the infectivity of clinical specimens (26, 27).

As expected, specimens containing high copy numbers of the viral genome (suggesting high viral loads) tended to be culturable compared with those with fewer genome copies. A previous study indicated that 6 log_10_ genome copies/ml sample might be required for virus isolation based on analysis of a series of specimens from patients hospitalized for COVID-19, and no virus could be isolated from specimens collected after day 8 of illness irrespective of the high viral loads (9). The lowest genome copy number detected in our culturable specimens was 5.4 log_10_ genome copies/ml sample of the *nsp12* gene. This difference from the previous study might be related to differences in experimental conditions and laboratories. Thus, 5 to 6 log_10_ genome copies/ml sample seems to be a reasonable viral load required for virus isolation. However, more and systematically collected specimens should be compared in the future to validate this prediction. Another feature associated with culturable specimens was the strong linear correlation between copy numbers of structural and nonstructural genes, indicating that the viral genome of cultural specimens was intact, possibly reflecting an infectious virion. In contrast, the considerably higher RNA level of structural genes detected in nonculturable specimens might reflect the presence of replication intermediates retained in epithelium cells (21), while the nonculturable specimens characterized by highly nonstructural genes might contain viral genomes yet to be degraded. For specimens containing high viral loads with a high correlation among genes, the virus can be inactivated by neutralizing antibodies that might cause aggregation of the virus to prevent nucleic acid degradation (14). This hypothesis was supported by the finding that seroconversion occurred 7 to 14 days after symptom onset when no rapid decline in viral load was observed (9).

Overall, this study provides evidence that the infectiousness of clinical specimens from COVID-19 patients can potentially be determined by both the SARS-CoV-2 gene copy numbers and genome integrity. Nucleic acid detection is undoubtedly valuable in detecting SARS-CoV-2; however, other serological tests should be performed in parallel to best evaluate the disease course of a COVID-19 patient as demands of health care systems are robustly increasing due to the pandemic.

## Supplementary Material

Supplemental file 1

Supplemental file 2
