# Supplementary material for: Culture-Based Virus Isolation To Evaluate Potential Infectivity of Clinical Specimens Tested for COVID-19
Source: J Clin Microbiol. 2020 Jul 23;58(8):e01068-20. doi: 10.1128/JCM.01068-20 (PMC7383522; doi:10.1128/JCM.01068-20)
Supplement: Supplemental file 1 [file JCM.01068-20-s0001.pdf]

SUPPLEMENTAL TABLE 1. Cell culture-based virus isolation of clinical specimens used in this study.

Case no	Specimen type	Duration 1 (onset to collection)	Duration 2 (collection to inoculation)	*nsp 12* (Ct)	*E* (Ct)	*N* (Ct)	Culture days
**Culturable samples with one freeze-thaw cycle (n = 5)**							
1-2	SP	3	22	19.38	18.68	26.86	10
2	NP	N/A	13	29.87	28.74	N/A	14
6[a]	NP	5	3	22.8	22.13	28.49	18
11	OP	3	1	21.41	20.2	N/A	9
12[a]	OP	N/A[b]	2	25.35	24.12	28.09	18
**Culturable samples without freeze-thaw cycle (n = 18)**							
16	NP	1	1	18.94	18.36	22.14	9
17	NP	2	3	21.7	20.9	26.06	4
18	NP	5	3	22.63	19.94	23.67	4
19	OP	1	2	24.5	24.08	28.81	4
20	OP	2	2	25.7	22.76	28.1	4
23	OP	6	1	25.04	24.04	26.9	5
25	OP	3	1	23.7	21.14	24.89	5
26	OP	11	1	21.84	19.38	23.13	5
27[a]	NP	N/A[b]	2	27.7	25.51	31.05	5
31	OP	4	1	24.53	22.13	25.63	5
33	OP	1	2	21.23	19.8	25.65	3
34	OP	6	2	31.47	31.46	32.81	3

Case no	Specimen type	Duration 1 (onset to collection)	Duration 2 (collection to inoculation)	*nsp 12* (Ct)	*E* (Ct)	*N* (Ct)	Culture days
36-1	NP	3	2	27.96	27.05	32.31	3
36-2	SP	3	2	17.75	16.85	22.73	3
38-1	OP	1	2	19.69	18.85	24.76	3
38-2	NP	1	2	20.81	19.9	25.17	3
42	OP	1	2	26	25.51	30.54	2
45	NP	1	3	29.6	23.5	35.2	7
**Non-culturable samples with one freeze-thaw cycle (n = 11)**							
1-1	NP	3	22	28.39	30.55	33.34	>15
3-1[a]	NP	10	19	28.9	25.05	30.08	>28
3-2	SP	11	23	34.43	31.8	34.36	>23
3-3	SP	11	21	N/D	32.93	36.45	>23
3-4	OP	26	7	N/D	34.9	42.47	>23
4[a]	OP	N/A[b]	2	N/D	38.33	N/D	>26
5	OP	1	8	36.28	31.91	N/D	>20
7[a]	NP	8	7	28	26.61	26.61	>15
8[a]	NP	9	7	25.6	28.77	28.77	>20
9[a]	NP	2	7	23.92	26.39	26.39	>20
10[a]	NP	2	7	30.77	29.85	32.29	>20
**Non-culturable samples without freeze-thaw (n = 26)**							
13-1[a]	OP	7	4	26.7	27.48	31.73	>15

Case no	Specimen type	Duration 1 (onset to collection)	Duration 2 (collection to inoculation)	*nsp 12* (Ct)	*E* (Ct)	*N* (Ct)	Culture days
13-2	OP	24	2	31.63	32.47	34.28	>19
14[a]	OP	N/A	1	27.28	26.59	32.04	>19
15-1[a]	NP	N/A	2	26.02	24.95	29.73	>14
15-2	SP	N/A	2	22.32	20.89	26.89	>28
21[a]	OP	1	1	28.03	26.27	30.52	>23
22	OP	3	1	26.74	26.3	29.55	>23
24	OP	11	1	32.92	28.94	32.54	>21
28	OP	4	1	33.83	28.59	36.41	>21
29	OP	10	1	30.53	29.76	32.41	>21
30-1	OP	3	1	36.52	30.72	33.78	>21
30-2	SP	3	1	33.53	29.86	32.14	>21
32	OP	9	1	34.95	30	N/D	>21
35	NP	9	1	26.14	25.21	30.7	>28
37	OP	5	2	30.88	34.97	N/D	>19
39-1	OP	1	2	26.57	25.52	30.05	>19
39-2	SP	1	2	28.69	27.93	31.53	>19
40	OP	0	4	23.47	22.61	28.47	>21
41	NP	8	3	35.6	35.45	N/D	>21
43	OP	1	2	28.5	27.95	32.25	>21
44	NP	13	3	35.5	36.31	N/D	>21

Case no	Specimen type	Duration 1 (onset to collection)	Duration 2 (collection to inoculation)	*nsp 12* (Ct)	*E* (Ct)	*N* (Ct)	Culture days
46	NP	2	2	29.23	29.11	32.98	>19
47	NP	1	5	25.67	24.69	28.22	>28
48	NP	8	1	25.89	24.39	28.97	>28
49[a]	NP	2	2	25.03	25.88	28.73	>28
50	NP	0	2	26.3	30.1	31.4	>28

[a]Retrospective cases.

[b]Not applicable, asymptomatic cases.

Abbreviations: NP, nasopharyngeal swab; SP, sputum; OP, oropharyngeal swab; Ct, cycle threshold; N/A, not applicable; N/D, not detectable.
