# Supplementary material for: Culture-Based Virus Isolation To Evaluate Potential Infectivity of Clinical Specimens Tested for COVID-19
Source: J Clin Microbiol. 2020 Jul 23;58(8):e01068-20. doi: 10.1128/JCM.01068-20 (PMC7383522; doi:10.1128/JCM.01068-20)
Supplement: Supplemental file 2 [file JCM.01068-20-s0002.pdf]

SUPPLEMENTAL TABLE 2. Raw data of real-time RT-PCR assessing serially diluted *nsp12*, *E*, and *N* standard RNA.

Gene	Copies	Log_{10} copies/reaction	Ct	Estimated Log_{10} copies/mL[a]
nsp12	50000000000	10.70	5	12.30
	5000000000	9.70	8.03	11.30
	500000000	8.70	12.69	10.30
	50000000	7.70	17.08	9.30
	5000000	6.70	21.09	8.30
	500000	5.70	24.67	7.30
	50000	4.70	26.93	6.30
	5000	3.70	28.02	5.30
	500	2.70	N/D	4.30
	50	1.70	N/D	3.30
	5	0.70	N/D	2.30
E	50000000000	10.70	4.69	12.30
	5000000000	9.70	9.09	11.30
	500000000	8.70	13.83	10.30
	50000000	7.70	18.78	9.30
	5000000	6.70	23.21	8.30
	500000	5.70	26.78	7.30
	50000	4.70	30.48	6.30
	5000	3.70	32.67	5.30
	500	2.70	33.13	4.30

	50	1.70	35.45	3.30
	5	0.70	N/D	2.30
N	50000000000	10.70	7.34	12.30
	5000000000	9.70	14.13	11.30
	500000000	8.70	18.32	10.30
	50000000	7.70	22.16	9.30
	5000000	6.70	28.07	8.30
	500000	5.70	29.26	7.30
	50000	4.70	32.21	6.30
	5000	3.70	36.02	5.30
	500	2.70	36.95	4.30
	50	1.70	N/D	3.30
	5	0.70	N/D	2.30

[a] A dilution factor of 40 is used to convert the copies per reaction to copies per mL.

Abbreviations: Ct, cycle threshold; N/D, not detectable.
